# Role of TNF–TNF Receptor 2 Signal in Regulatory T Cells and Its Therapeutic Implications

**DOI:** 10.3389/fimmu.2018.00784

**Published:** 2018-04-19

**Authors:** Sujuan Yang, Julie Wang, David Douglass Brand, Song Guo Zheng

**Affiliations:** ^1^Department of Clinical Immunology, Third Hospital at Sun Yat-sen University, Guangzhou, China; ^2^Division of Rheumatology, Milton S. Hershey Medical Center at Penn State University, Hershey, PA, United States; ^3^Research Service, Memphis VA Medical Center, Memphis, TN, United States

**Keywords:** tumor necrosis factor α, tumor necrosis factor receptor 1, tumor necrosis factor receptor 2, regulatory T cells, autoimmune diseases

## Abstract

Tumor necrosis factor α (TNFα) is a pleiotropic cytokine which signals through TNF receptor 1 (TNFR1) and TNF receptor 2 (TNFR2). Emerging evidence has demonstrated that TNFR1 is ubiquitously expressed on almost all cells, while TNFR2 exhibits a limited expression, predominantly on regulatory T cells (Tregs). In addition, the signaling pathway by sTNF *via* TNFR1 mainly triggers pro-inflammatory pathways, and mTNF binding to TNFR2 usually initiates immune modulation and tissue regeneration. TNFα plays a critical role in upregulation or downregulation of Treg activity. Deficiency in TNFR2 signaling is significant in various autoimmune diseases. An ideal therapeutic strategy for autoimmune diseases would be to selectively block the sTNF/TNFR1 signal through the administration of sTNF inhibitors, or using TNFR1 antagonists while keeping the TNFR2 signaling pathway intact. Another promising strategy would be to rely on TNFR2 agonists which could drive the expansion of Tregs and promote tissue regeneration. Design of these therapeutic strategies targeting the TNFR1 or TNFR2 signaling pathways holds promise for the treatment of diverse inflammatory and degenerative diseases.

## Introduction

Tumor necrosis factor α (TNFα) is an essential signaling protein in the innate and adaptive immune systems. It also plays an important role in tissue degeneration and repair (1). It is now recognized that the expression of TNF receptor 2 (TNFR2) is more limited than that of TNF receptor 1 (TNFR1). In addition, new evidence suggests that the sTNF-mediated signaling pathway *via* TNFR1 drives a predominantly pro-inflammatory program whereas mTNF binding to TNFR2 primarily initiates immune modulation and tissue regeneration. These findings suggest that we may selectively target TNFR1 and TNFR2 for therapeutic purposes, providing promise for the context-specific treatment of autoimmune diseases. This review is provided to summarize TNFα and TNFR expression, structure, and signaling pathways, to discuss TNFR1/TNFR2 signaling in autoimmune diseases especially concerning their correlation with Tregs and organ regeneration, as well as to propose treatment strategies aimed at TNFR1/TNFR2 in autoimmune diseases.

## The Basic Biology of TNFα and TNFR

### Expression, Structure, and Function of TNFα

Tumor necrosis factor α plays a vital role in many physiological and pathological conditions. First, TNFα is essential for the regulation of embryonic development, the sleep–wake cycle, lymph node follicle, and germinal center formation. Second, TNFα not only promotes the production of inflammatory cytokines but also enhances the adhesion and permeability of endothelial cells and promotes the recruitment of immune cells such as neutrophils, monocytes, and lymphocytes to sites of inflammation (2, 3). These actions help to mediate both acute and chronic systematic inflammatory reactions under conditions of infection or autoimmunity. In addition, TNFα also causes cell apoptosis and necrosis under specific conditions. Furthermore, high levels of TNFα can also result in cachexia and endotoxin-induced septic shock (4). It has also been identified as an endogenous pyrogen.

Tumor necrosis factor α is primarily generated by macrophages and monocytes. However, other cells such as some subsets of T cells, NK-cells, dendritic cells, B cells, cardiomyocytes, fibroblasts, and astrocytes are also the producers of this cytokine at a low level (5, 6).

Tumor necrosis factor α is a type II transmembrane protein. It exists as a membrane-bound form (mTNFα) with relative molecular weight 26 kDa primarily. mTNFα can be processed into 17 kDa soluble TNFα (sTNFα) through the action of the matrix metalloproteinase known as TNFα converting enzyme (TACE: ADAM17) (7, 8). In addition, mTNFα also has the ability to process external signals as a receptor (9). sTNFα circulates throughout the body and confers TNFα with its potent endocrine function, far away from the site of its synthesis. Both sTNFα and mTNFα are active as non-covalently bonded homotrimers.

While bacterial lipopolysaccharide (LPS) serves as a major stimulant of the innate immune system, microbial antigens, enterotoxins, and cytokines including TNFα itself are also able to trigger TNFα production. TNFα also stimulates the generation of numerous pro-inflammatory cytokines including IL-6, IL-8, TNFα itself, adhesive molecules, chemokines, and metalloproteinases (10, 11), potentially leading to a TNFα-mediated pro-inflammatory autocrine loop (12). On the other hand, TNFα can boost the synthesis of anti-inflammatory factors such as IL-10 and corticosteroids, to limit the inflammatory cytokines secretion. As a whole, TNFα initiates a rapid and vigorous immune reaction, thus limiting the extent and duration of inflammation when the invasion has been resolved (13). Furthermore, serving as a co-stimulator, TNFα enhances the reactions of neutrophils, monocytes, and lymphocytes for defense against microbes.

### Expression, Structure, and Signaling Pathways of TNFR

Tumor necrosis factor α exerts its function *via* two different type I transmembrane receptors, TNFR1 and TNFR2. Each has a characteristic extracellular domain, a transmembrane segment, and intracellular domain. The extracellular domains of both receptors have similar a cysteine-rich motif that is repeated two to six times, are active as homodimers but intriguingly do not form TNFR1/TNFR2 heterodimers (14). Nevertheless, the intracellular segments of TNFR1 and TNFR2 do not bear homologous sequences and activate distinct signaling pathways (15).

Both TNFR1 and TNFR2 membrane receptors also can be converted into soluble forms (sTNFR1 and sTNFR2) through the activity of TACE enzymes. Both TNFRs can interact with either mTNFα or sTNFα. TNFR1 is ubiquitously expressed on nearly all cells in the body and can be activated by both mTNFα and sTNFα. TNFR2, conversely, is restricted to thymic T lymphocytes, endothelial cells, microglia, and oligodendrocytes (16), and can only be fully initiated by mTNFα. Once mTNFα binds to TNFR2, the combination is too stable to dissociate (17). This is not the case for sTNFα which induces weak signaling and exhibits a low affinity for TNFR2 (18). Other salient features of TNFR2 are that cellular activation status highly regulates its expression and unlike TNFR1, it does not contain a cytoplasmic death domain.

**Figure 1:**
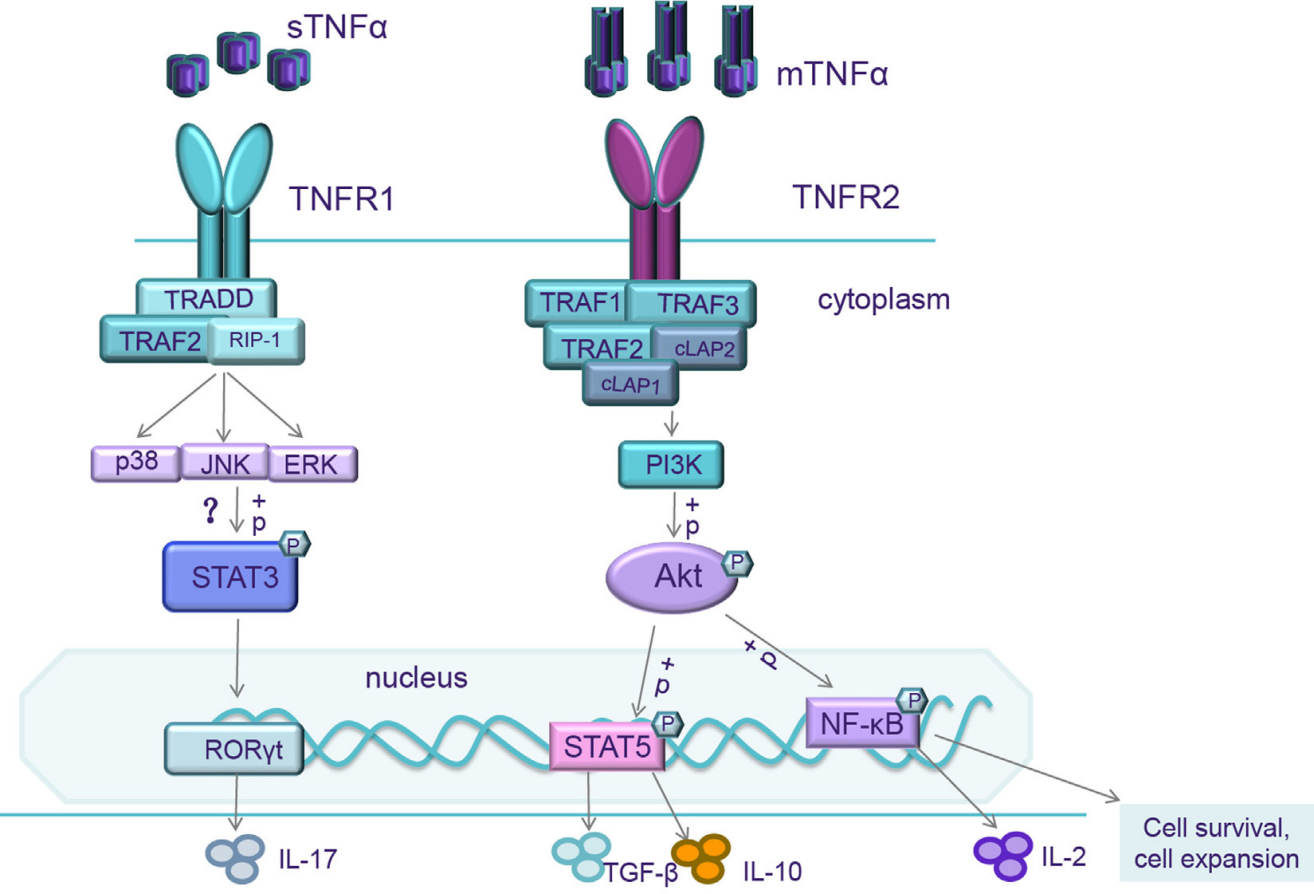
When mTNF/TNF receptor 2 (TNFR2) is activated, the intracellular domains recruit existing cytoplasmic TNF receptor-associated factor-2 (TRAF-2)–cIAP-1–cIAP-2 complexes resulting in the initiation of both canonical and non-canonical NF-κB/Rel and MAPK pathways activation. NF-κB/Rel and MAPK pathways activate IL-2 promoter and trigger IL-2 expression. NF-κB pathways also transcript genes associated with cell survival and cell proliferation. So, mTNF/TNFR2 signaling can enhance expansion and stability of Tregs and increase Treg sensitivity to low level of IL-2. It also activates the reciprocal PI3K/Akt pathway. Activation of Akt signaling impairs Th17 differentiation, correlated with an increased phosphorylation of STAT5 (143). When soluble TNFα (sTNFα)/TNFR1 is activated, the intracellular domains interact with TRADD, which receptor interacting protein-1 (RIP-1) and TRAF-2 to form Signal complex I, then further triggering extracellular signal-regulated kinases (ERKs), p38, and c-Jun N-terminal kinase (JNK). The mechanisms of TNFR1 on Th17 differentiation are still unclear. These transcription factors might phosphorylate STAT3, upregulate the level of ROR-γt, and increase IL-17 production.

It is well accepted that TNFα binding to TNFR1 activates two different intricate signal pathways: the maintenance of cell survival and the promotion of inflammatory cytokine expression; cell apoptosis and necrosis. The balance between these two pathways hinges upon many factors such as cell type, cell activation status, an intracellular or extracellular microenvironment, recruitment of adaptor molecules, the concentration of complex inhibitors of apoptosis proteins (cIAP), or the level of NF-κB expression (19). When TNFα binds to TNFR1, the intracellular domains interact with TNFR type 1-associated death domain protein (TRADD), which recruits receptor interacting protein-1 (RIP-1) and TNF receptor-associated factor-2 (TRAF-2) to form Signal complex I (5) (Figure 1). Signal complex I can trigger NF-κB, which launches the transcriptions of many different genes including those associated with cell survival, production of inflammatory cytokines, and antiapoptotic gene pathways. Signal complex I is also able to activate extracellular signal-regulated kinases, the stress-activated MAP kinases p38, and c-Jun N-terminal kinase (JNK), which are important for AP-1, the important promoter of inflammation and proliferation, and other transcription factors through MAPK3 signaling pathways (20–22).

Signal complex I formation is temporary and rapidly dissociates from TNFR1, mediating the binding of the Fas-associated death domain protein (FADD) to form Signal complex II which coordinates downstream signaling of the caspase cascade (23). When the kinase activities of RIP-1 and RIP-3 inhibit apoptosis signaling, necrosis is activated (24).

Recently, several studies have demonstrated that TNFR2 promotes a remarkable degree of cell activation, migration, and proliferation (24). When TNFα binds to TNFR2, the intracellular domains recruit existing cytoplasmic TRAF-2–cIAP-1–cIAP-2 complexes (25) (Figure 1). cIAP can exert ubiquitin-ligase activity and can inhibit caspases and other apoptosis-inducing factors (5), resulting in the initiation of both canonical and non-canonical NF-κB activation (25–27). The interaction of TNFα with TNFR2 also activates the reciprocal PI3K/Akt pathway. This pathway not only maintains survival and enhances proliferation (28, 29) but also recruits Etk and forms the TNFR2–Etk–VEGFR2 (vascular endothelial growth factor receptor 2) complex which participates in cell adhesion, migration, survival, and proliferation (30, 31).

Although TNFR2 triggers NF-κB in a slower manner than TNFR1, TRFR1 maintains a longer duration of NF-κB activity (25). Even though TNFR2 lacks a death domain, caspase activation and cell apoptosis can be initiated under conditions of stress or when the cIAP pool exhausted *via* interaction of intracellular domains with Signal complex II. Other theories suppose that TNFR2 activation exhausts the cIAP pool, which facilitates a shift of TNFR2 signaling to FADD, triggering the apoptosis pathway.

## The Function of TNFR1 and TNFR2 on Autoimmune Diseases

Disease models using transgenic mice have broadened our horizons concerning the importance of pathogens in triggering or shaping autoimmunity. Compared with wild-type mice, TNFα^−/−^ mice exhibit an enhanced susceptibility to pathogen invasion (2). They also exhibit a deficiency in TNFR1 (32). It is noteworthy that mice expressing non-cleavable TNF (which cannot be processed into sTNF) have a diminished capacity to resist pathogens (33, 34). This demonstrates that many of the pro-inflammatory functions of sTNF are indeed mediated by TNFR1 signaling, while mTNF (predominantly *via* TNFR2) can at least partly provide the immune system with some pathogen protection.

Overexpression of TNF results in a severe chronic inflammatory arthritis in collagen-induced arthritis (CIA) mice, an animal model of rheumatoid arthritis (RA). When the TNFR1 gene is knockout, these arthritic effects are largely diminished, whereas TNFR2 deficiency exacerbates disease (35, 36). Interestingly, the use of TNF inhibitors dramatically improves symptoms in a manner reminiscent of TNFR1 deficiency (36, 37). In addition, central nervous system-specific overexpression of TNF in transgenic mice also resulted in a spontaneous severe demyelination (38). These results confirm the pro-inflammatory role of TNFR1, while leaving the door open for an immune-modulatory role of TNFR2. In the experimental autoimmune encephalomyelitis (EAE) mouse model, TNF knockout delayed disease onset, however, once established, the symptoms were more serious in knockouts than in wild-type mice (39). This raises the suggestion that TNF is essential to the promotion of a potent immune response *via* TNFR1. However, once the immune response is triggered, the absence of TNF may result in the failure to expand and activate Tregs *via* TNFR2 which in turn results in tissue and organ damage. Increasing evidence indicates that TNFR2 plays a vital role in the modulation of the immune system, most likely through its interactions with Tregs.

It has been well studied that polymorphisms in the TNFR2 gene have a strong correlation with a wide variety of autoimmune diseases, e.g., RA (40–42), Crohn’s disease (43), systemic lupus erythematosus (44), ankylosing spondylitis (AS) (45), inflammatory bowel diseases (IBD) (46), and ulcerative colitis and scleroderma (47). The consequence of this polymorphism is to hamper TNF binding to TNFR2, which subsequently limits the activation of NF-κB (48), and most likely hampers TNFR2 signaling pathway in Tregs.

## TNFR2’s Relationship with Tissue Regeneration and Tregs

### The Relationship Between TNFR2 and Tissue Regeneration

TNF receptor 2 provides a critical contribution to neural survival and regeneration. In the mouse model of retinal ischemia, TNFR2 showed a protective function by activating the Akt signaling pathway (49). The cuprizone-induced demyelination and remyelination mouse model gave similar results. In this model, TNF or TNFR2 knockout led to delayed remyelination and a decreased proliferation and maturation of oligodendrocyte progenitors. These findings provide support for the notion that TNF/TNFR2 serves as principal players in oligodendrocyte regeneration (50). A tissue regenerative role for the TNFR2 signaling pathway has also been described in several other disorders (51). Several other studies have also indicated that TNFR2 agonists are active in pancreatic regeneration, cardioprotection, remyelination, and survival of some neuron subtypes and also in stem cell proliferation (51–54).

### The Relationship Between TNFR2 and Tregs

The interplay between inflammatory and regulatory pathways orchestrates an effective immune response that provides protection from pathogens while limiting injury to host tissue. Tregs are prototypical immunosuppressive cells that dampen excessive immune responses and maintain immune homeostasis by inhibiting effector T cell proliferation and cytokine production which prevents the development of autoimmune diseases and tissue destruction (55–58). Regulatory T cells can mediate their suppressive function either by secreting cytokines like IL-10, TGF-β, or IL-35 or by direct cell–cell contact (59, 60). These cells can act by suppressing the effector T cells directly at the target site (61), by suppressing DC in the regional lymph nodes and thereby preventing priming of T cells in the regional lymph nodes (62), or by recruiting mast cells to the site (63). Mice deficient in Foxp3^+^ T cells develop fatal autoimmune disease (64), and continuous expression of Foxp3 throughout life prevents autoimmunity (65). Recent studies have contradictorily demonstrated that TNF upregulates or downregulates the expansion and function of Tregs *via* TNFR2.

One specific study has demonstrated that as with human Tregs, both thymic and peripheral murine CD4^+^CD25^+^ Tregs expressed remarkably high levels of TNFR2 relative to CD4^+^CD25^−^ effector T cells (66). By contrast, TNFR1 was barely detectable.

When responding to TCR stimulation, TNFR2 expression on Tregs is further increased relative to activated effector T cells (67). In TNFR2 knockout mice, although the numbers and function of Tregs are comparable with wild-type mice, these Tregs failed to expand when stimulated under inflammatory conditions either *in vivo* or *in vitro*. This suggests that under non-inflammatory conditions, TNF is not required for thymic Tregs to maintain immune homeostasis (67, 68). Conversely, TNFR2 can mediate the activation of anergic Tregs in response to TCR stimulation (67), having profound effects on their stabilization (69), proliferation (70), and function (71).

Interestingly, one study about type 1 diabetes model on NOD mice found that TNFR1 deficiency protected the mice from diabetes and showed mild peri-insulitis. The absence of TNF–TNFR1 signaling increased the number and function of Tregs both *in vitro* and *in vivo* (72). They proposed that the primed effector T cells secreting TNF that signals through TNFR2, which is constitutively expressed at a high level on Tregs (67), leads to expansion of Tregs. We also considered the TNFR1 deficiency is consequential to elevated TNFR2 signaling on Tregs, as a result of increased ligand availability as opposed to a loss of stimulatory TNFR1 signaling. As a consequence, increased Tregs prevent effector T cells migrating into islets. Similar work has been exhibited in EAE model (73).

We and other investigators have reported that IL-2 is essential for the development and maintenance of Foxp3^+^ Treg cells (56, 57). Neutralization of circulating IL-2 elicits autoimmune gastritis in BALB/c mice and triggers early onset of diabetes by inhibiting physiological proliferation of peripheral CD4^+^CD25^+^ cells, but not CD4^+^CD25^−^ cells (74). Interestingly, TNF can enhance this effect markedly both in human and mice (67, 75). In mice, it exerts the effect markedly in a time-dependent and dose-dependent manner (67). The initial exposure to TNF transiently abrogates Treg suppressive functions, whereas longer exposures restore their suppressive activity. This means that short-term stimulation of TNF mimics the early phases of the inflammatory reaction, thus allowing effector T cells to escape from the inhibition mediated by Tregs, presumably favoring elimination of the pathogens. However, long-term exposure to TNF may facilitate the activation and expansion of Tregs, restoring their suppressive capabilities, thus limiting excessive inflammation.

**Figure 2:**
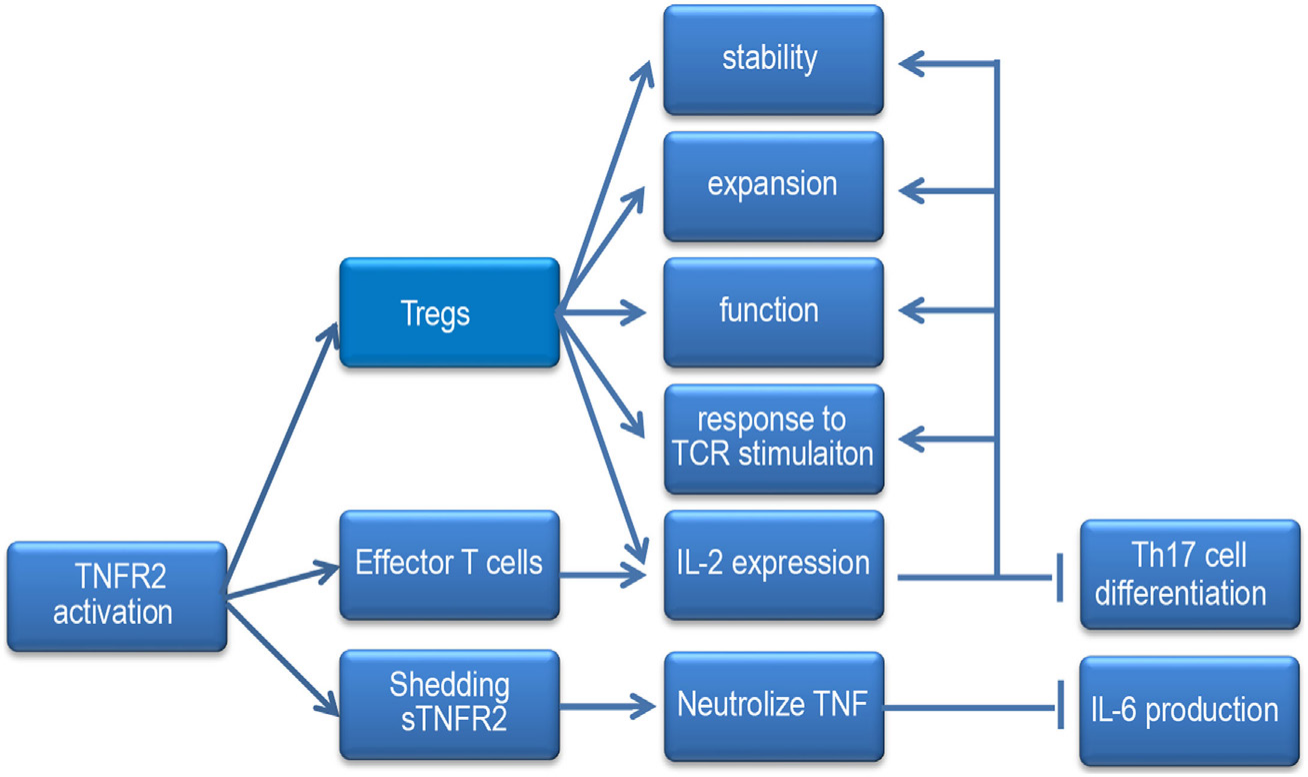
When the TNF receptor 2 (TNFR2) signaling pathway is activated, it increases Tregs stability, responses to TCR stimulation, expansion, and function. It enhances Tregs and effector T cells to produce IL-2 and promotes the sensitivity of Tregs to IL-2. IL-2 can inhibit Th17 cells differentiation and the effect of TNFR2 signaling on Tregs. Under inflammatory condition, mTNFR2 can shed to sTNFR2, sTNFR2 neutralizes TNF and hampers IL-6 expression.

Recently, one investigation showed that anti-TNF antibody, adalimumab expanded the pool of Tregs and maintained their function *via* mTNF/TNFR2 both *in vitro* and *in vivo*. Moreover, upregulation of Foxp3 by adalimumab was reliant upon low levels of IL-2 production and subsequently STAT5 activation of Tregs (76). They proposed that TNFR2 is able to increase the sensitivity of IL-2 signaling, thereby amplifying the impact of small changes in IL-2 production (77). Coincidentally, another study demonstrated that IL-2 transcription was directly triggered by TNFR2. It showed that CD4^+^ T cell intrinsic tmTNF/TNFR2, but not sTNF/TNFR1, promotes Il2 promoter activity and IL-2 mRNA stability in a Foxp3-independent manner both *in vitro* and *in vivo*. When tmTNF/TNFR2 signaling is blocked or impaired, IL-2 production is reduced, and the Th17 differentiation elevated, which was associated with increased STAT3 activity and ROR-γt level, decreased STAT5 activity, while, it can be prevented by adding exogenous IL-2 (78). However, whether TNFR2 regulates IL-2 expression in a Foxp3-independent manner or a Foxp3-dependent manner, whether IL-2 can in turn regulate TNFR2 expression, the precise signaling pathways by which TNFR2 regulates IL-2 expression still remain important areas for future studies (Figure 2).

Work in the last decade has established that the subset of Foxp3^+^ Tregs expressing TNFR2 showed increased suppressive function relative to those that did not express TNFR2. However, these studies suggested that Foxp3 expression may not be the only factor to confer Treg suppressive capacity (79). Indeed, TNFR2 may be needed as a unique activator to maximize their suppressive activity (67, 79). Furthermore, when TNFR2^−/−^ mice are used in the colitis model, some studies have found that nTregs require TNFα *via* TNFR2 as a critical factor for optimizing Treg suppressive function under inflammatory conditions whereas iTregs are fully suppressive without TNF signaling (79). Therefore, it is likely that anti-TNFα therapy for different human autoimmune diseases may have dichotomous effects on the function of nTregs versus iTregs. Whether or not this has any influence on disease expression would depend on whether nTregs or iTregs play the predominant regulatory role in that specific disease. Diseases in which iTregs are functionally predominant would encourage the anti-inflammatory effects of anti-TNFα therapy, with no deleterious effects on iTregs and a favorable response. By contrast, diseases in which nTregs functionally predominate, anti-TNFα therapy might result in a loss of Treg function. This dichotomy offers a novel mechanistic paradigm for the enigma of variable responses to anti-TNF-α therapy in different human diseases.

It is well accepted that Tregs consist of two major identified subtypes: natural or thymic Tregs (nTregs/pTregs), developed in the thymus; induced Tregs or peripheral Tregs (iTregs/pTregs) generated in the periphery from CD4^+^CD25^−^ T cells (pTregs) or iTregs induced with anti-CD3/CD28 coated beads, IL-2 and TGF-β from naive CD4^+^ T cells *in vitro* (iTregs) (80–82). The two populations have subtle difference, such as methylation status of conserved non-coding sequence 2 [known as the Treg cell-specific demethylated region (TSDR) in the Foxp3 locus]. Although the stability of Tregs is still controversial, most researchers generally recognized that nTregs are predominantly more stable and long-lived than iTregs because TSDR in nTregs but not iTregs is hypomethylated, which is great important for Foxp3 stability (83, 84). While, a small percentage of nTregs may become unstable, losing Foxp3 expression and transforming to effector T cells, such as Th1, Th17 cells under some pathogenic conditions. As stated earlier, the suppressive function of Foxp3^+^ Tregs expressing TNFR2 was superior to those that did not express TNFR2 (79). Okubo et al. found one method for Tregs expansion *ex vivo* using a synthetic TNFR2 agonist which produces Tregs with 14 homogenous cell-surface markers (85). Although it still needs more researches to definite this issue, the relationship between TNF–TNFR2 and nTregs provides a promising way to apply Tregs to autoimmune therapy. Even if some researchers insisted that iTregs induced by IL-2 and TGF-β is less stable, we and others found these iTregs are more stable and resistant to phenotypic plasticity in some autoimmune diseases and acute graft-versus-host disease (58, 86–90). Furthermore, our laboratory recently found that TNF (*via* TNFR2) enhanced the differentiation and suppressive function of iTregs induced *in vitro* (unpublished observation). As such, therapeutic intervention with these iTregs *via* TNFR2 has become a promising strategy for the treatment of autoimmune disorders (91).

Intriguingly, van Mierlo et al. described that CD4^+^CD25^+^ Treg cells were able to shed higher amounts of TNFR2 for a longer period of time than CD4^+^CD25^−^ T cells. In addition, WT Tregs can suppress IL-6 production when LPS was injected into mice. By contrast, TNFR2-deficient Tregs failed to do this but maintained their suppressive function *in vitro* (92). Thus, shedding of TNFR2 might represent yet another novel mechanism for Tregs to inhibit the pro-inflammatory action of TNF at inflammatory sites. It was presumed that low concentrations of TNFR2, through receptor shedding or other processes, might possibly decrease the concentration of TNF and prevent it from binding to inflammatory cells (93, 94). Unfortunately, this concept highlights a discrepancy. If CD4^+^CD25^+^ Tregs shed enough of their TNFR2, TNF will fail to have any effects on Treg activation, expansion, or function.

Despite this question, some laboratories have offered conflicting results concerning the effect of TNF on human Tregs. They believe that TNF stimulation directly hampers the suppressive capacity of human CD4^+^CD25^high^ Tregs both *in vitro* and *ex vivo* (71, 95–98). Moreover, the inhibitory effects of TNF are more apparent under coculture conditions than they are under pretreatment condition (99). This discrepancy may be due to the cross-species differences between murine and human T cells. It also raises the possibility of differences in the sensitivity of the experimental conditions or the differing methodologies employed in each laboratory. Valencia et al. showed that CD4^+^CD25^high^ Tregs stimulated with high levels of TNF and IL-2 lost their immune suppressive capabilities, presumably these findings were as a result of only the early stages of TNF stimulation (95).

Indeed, these studies also showed that neutralization of TNF might actually restore Treg suppressive ability and maintain the survival of Tregs in patients with RA and IBD (95, 98, 100, 101). In neonatal NOD mice, treatment with TNF promoted the development of diabetes accompanied with a reduced number and impaired function of Tregs instead (102). By contrast, administration of TNF to young adult NOD mice also ameliorated diabetes but enhanced the proliferation of Tregs (102) instead. We hypothesized that the NOD mouse is a spontaneous animal model of T1DM. The severity of inflammation is deteriorating with age in NOD mice, and the immune microenvironment is changing, which may impact the density of TNFR1 and TNFR2 on the surface of Tregs, the sensitivity to TNF, even the TNF form tending to sTNF or mTNF, and the activation of TNFR1 or TNFR2 signaling pathways. Nonetheless, the precise effect of TNF on Tregs activity remains elusive, and it still needs more in-depth investigations.

## Targeting sTNF/TNFR1 in Autoimmune Diseases

Treatment with TNF inhibitors has been a successful strategy for several diseases such as RA, IBD, psoriasis, and cancer-related cachexia. Recent anti-TNF therapies are all aimed at directly binding the ligand to TNF. Five anti-TNF drugs are currently approved for the therapy of human autoimmune disorders: RA, plaque psoriasis, psoriatic arthritis, AS, and IBD. Trade names for these drugs include infliximab, adalimumab, certolizumab pegol, golimumab, and etanercept (103–107). Notably, it raises a novel mechanism that adalimumab prefers binding to mTNF on monocytes and increased their mTNF expression, followed by enhancement of Treg TNFR2 expression and its binding to mTNF. As a consequence, adalimumab expanded functional Tregs equipped to suppress Th17 cells (76).

Despite the wide use of TNF inhibitors, drawbacks include severe side effects like opportunistic infections, reactivation of tuberculosis, and even development of autoimmune diseases, lymphoma, and many other cancers (108–111). In addition, some patients do not respond well to these anti-TNF treatments (105, 106). Furthermore, in clinical trials on MS patients, treatment with TNF inhibitors resulted in disease exacerbation (112, 113).

Because sTNF/TNFR1 may play a role in promoting inflammation, and because mTNF/TNFR2 can result in immune modulation and tissue regeneration, new therapeutics selectively targeting sTNF/TNFR1 have emerged. Both TNFR1-selective antagonists and sTNF-special antagonists may leave the mTNF/TNFR2 signaling pathway intact, which may minimize or diminish the detrimental effects caused by TNF inhibitors. This provides protective TNF-mediated responses such as neural regeneration, survival, and immune modulation without promoting inflammation. Indeed, mTNF alone may be enough to form TNF-dependent secondary lymphoid organ structure and granulomas (114, 115), and to partially provide resistance to pathogens (116, 117), without causing any autoimmune diseases (114, 116).

### sTNF-Selective Dominant-Negative TNF (dnTNF) Derivatives

A novel type of TNF inhibitor called signaling-incompetent dnTNF derivatives was described in 2005 (118). This TNF mutein can rapidly and specifically inactivate sTNF through interaction with endogenous sTNF, followed by formation of mixed TNF heterotrimers, leaving mTNF unaffected. XPro1595, an improved version of this mutein, exhibited a profound ameliorating effect on EAE and inflammatory arthritis. XPro1595-treated animals were also less susceptible to infection (119, 120). In addition, relative to etanercept, XPro1595 treatment significantly delayed onset and more efficiently ameliorated EAE symptoms (121), even when applied at the disease peak period (122). Interestingly, XPro1595 administration increased the level of TNFR2 expression in the lesion area, illustrating that mTNF signaling *via* TNFR2 may indeed play a role in neural regeneration (123).

### TNFR1-Selective Antagonistic TNF

Some investigators have identified R1antTNF, a TNFR1-selective antagonistic mutant TNF from a phage display library (124). The affinity of R1antTNF to TNFR1 is comparable to that of the human wild-type TNF, and it does not interfere with TNFR2-mediated bioactivity. In two acute hepatitis models, R1antTNF significantly ameliorated liver injury as demonstrated by reduced serum levels of alanine aminotransferase and pro-inflammatory cytokines. This therapeutic effect of R1antTNF had an advantage over that of current TNF inhibitors (125). PEG-R1antTNF, another TNFR1-selective antagonistic mutant TNF, remarkably decreased morbidity, ameliorated disease symptoms, and improved demyelination in EAE mouse model. Furthermore, it significantly suppressed Th1 and Th17 cell activation and infiltration in the spinal cord (126).

### TNFR1-Specific Antibodies

Recently, one study compared the therapeutic effects of the TNFR1-specific antibody, DMS5540 with that of etanercept in the CIA model. Both reagents comparably suppressed disease progression. One difference noted was that etanercept administration increased effector T-cell activity, specifically in joints undergoing remission. This was not observed in mice treated with DMS5540 (127). These findings suggest that the immune regulatory function of TNFR2 is masked by traditional TNF inhibitors, like etanercept. It proves the hypothesis that selective targeting of TNFR1 not only inhibits autoimmune responses but also enhances Treg activity, making it a better choice for TNF therapy over standard TNF inhibitors (127).

## Targeting TNFR2 in Autoimmune Diseases

It remains to be verified whether selective inhibition of sTNF/TNFR1 proves enough to redirect the available TNF to TNFR2 for improving immune regulation and tissue regeneration. In support of this, mTNF/TNFR1 plays a role in neuron cell survival in under certain circumstances (128). On the other hand, sTNF probably activates TNFR2 in high TNFR2-expressing cells such as Tregs (128). It still remains to be determined whether mTNF signaling *via* TNFR2 is enough to activate Tregs and if additional activation of sTNF *via* TNFR2 can promote Treg activity. Most importantly, it is likely to have a narrow range of safe and effective dose since TNFR1 is expressed ubiquitously almost all types of cells throughout the body. Thus, selective TNFR2 agonists can provide yet another tissue-specific or cell-specific therapy for autoimmune disorders.

TNF receptor 2 agonists were engineered using point mutation (129). Treatment with these TNFR2 agonists has been successfully used for cancer therapy and also for research in immunology (130). As mentioned earlier, mTNF binds much greater affinity to TNFR2 than to sTNF itself (130). The investigators undertaking this line of research also found another small synthetic molecule that acted as trimer ligands which were similar to TNFR2 (131). Over time, additional literature indicates that TNF and TNFR2 agonists exert a tremendous effect on heart regeneration, bone marrow stem cells, and even neuron regeneration in murine models of Parkinson’s disease (132–134).

TL1A-Ig, a natural TNF-receptor superfamily member agonist is used as a novel method for the *in vivo* expansion of Tregs (135). Okubo et al. found another method for Treg expansion *ex vivo* using a synthetic TNFR2 agonist which produces Tregs with 14 homogenous cell surface markers (85). This method provides an optimal way to obtain sufficient quantities of Tregs use in autoimmune therapy.

### TNF Inducers

One well-known TNF inducer is the mycobacterium bacillus Calmette–Guerin (BCG) vaccine. Another one is the BCG equivalent, complete Freund adjuvant. Although BCG induction of TNF can interact with both TNFR1 and TNFR2, it may induce TNF production at low levels, thereby possibly boosting the expansion of Tregs (85), which can provide benefits for the treatment of autoimmune diseases. Newly synthesized TNF inducers improve the specificity for the TNFR2 receptor and may hold the promise for the treatment of type 1 diabetes (136).

### CD3-Specific Antibodies

As a distinctive cell-surface marker of T cells, CD3 antagonists are targeted to be mainly applied as immunosuppressive agents to protect against organ transplant rejection. While a CD3-specific antibody generally seen as an immunosuppressive agent, it may promote TNF generation and TNFR2 expression (137), and thus exert the similar effects as the TNFR2 agonist.

It is particularly noteworthy that the safety profile of TNFR2 agonists has not been well defined. Not all of TNFR2 agonists exert their effects using the same mechanism. Some can function as agonists while some TNFR2 antibodies can be antagonists and still others may induce anergy. It is clear that many different factors come into play concerning the activation and regulation of the balance between TNFR1 versus TNFR2 signaling. These subtle differences in TNF stimulation can result in the generation of divergent intracellular signaling pathways (138). More studies with these new agents are needed to determine if their use results in any changes in TNF signaling and what effects these changes have on physiological consequences.

To avoid the systemic toxicity, injection of TNFR2 agonists directly into sites of inflammation or lymphoid organs might be a promising approach. Their local application would be attractive for diseases such as autoimmune Sjogren’s syndrome or skin diseases because the skin is an easily accessible organ that is amenable to agonist delivery at desired concentrations, thus reducing systemic toxicity. However, not all lesions are as accessible. Pancreatic injection might potentially trigger pancreatitis.

In spite of their non-specificity, several alternative strategies are also in progresses which act *via* immune modulation of TNFR2 signaling pathways. However, high concentrations of TNFR2 agonists could potentially overwhelm TNFR2 signaling and might shift their activities to the pro-apoptotic pathway *via* TNFR1. This would result in apoptosis of both autoreactive and bystander cells. In spite of their lower relative toxicity to TNFR1 agonists, TNFR2 agonists might still have some degree of toxicity, particularly toward CNS cells (139). Overexpression of TNFR2 in a transgenic mouse model resulted in systemic toxicity (140) and also elicited several autoimmune diseases as mentioned earlier (141). It is noteworthy that one specific TNFR2 agonist enhanced thymocyte proliferation *in vitro* and *in vivo*, caused a febrile reaction and a transient inflammatory reaction (142). Thus, the potential toxicity of TNFR2 agonist therapy still needs to be investigated carefully.

## Conclusion

Taken together, as with anti-cytokine or immunosuppressive drug therapies which must be used continuously to keep steady blood concentration, any of the absolute TNF inhibitors, sTNF inhibitors, TNFR1 antagonists, or TNFR2 agonists, could potentially be given at a low dose discontinuously and intermittently. However, sTNF/TNFR1-special antagonists can specifically block TNFR1 signaling pathway and leave the protective effect, e.g., neural regeneration and immune modulation *via* TNFR2. Furthermore, TNFR2-special agonists avoid the detrimental effects initiated by total TNF inhibitors and sTNF/TNFR1-special antagonists due to their limited tissue expression. sTNF inhibitors, TNFR1 antagonists, and TNFR2 agonists, when used alone or in combination therapy, may provide a superior therapeutic strategy for the treatment of various autoimmune and degenerative diseases.

## Author Contributions

All authors listed have made a substantial, direct, and intellectual contribution to the work and approved it for publication.

## Conflict of Interest Statement

The authors declare that the research was conducted in the absence of any commercial or financial relationships that could be construed as a potential conflict of interest.

## References

[1] Baud V Karin M. Signal transduction by tumor necrosis factor and its relatives. Trends Cell Biol (2001) 11:372–7. 10.1016/S0962-8924(01)02064-5 11514191

[2] Pasparakis M Alexopoulou L Episkopou V Kollias G. Immune and inflammatory responses in TNF alpha-deficient mice: a critical requirement for TNF alpha in the formation of primary B cell follicles, follicular dendritic cell networks and germinal centers, and in the maturation of the humoral immune response. J Exp Med (1996) 184:1397–411. 10.1084/jem.184.4.1397 8879212 PMC2192824

[3] Mackay F Loetscher H Stueber D Gehr G Lesslauer W. Tumor necrosis factor alpha (TNF-alpha)-induced cell adhesion to human endothelial cells is under dominant control of one TNF receptor type, TNF-R55. J Exp Med (1993) 177:1277–86. 10.1084/jem.177.5.1277 8386742 PMC2190994

[4] Kothari N Bogra J Abbas H Kohli M Malik A Kothari D Tumor necrosis factor gene polymorphism results in high TNF level in sepsis and septic shock. Cytokine (2013) 61:676–81. 10.1016/j.cyto.2012.11.016 23317877

[5] Bradley JR. TNF-mediated inflammatory disease. J Pathol (2008) 214:149–60. 10.1002/path.2287 18161752

[6] Tracey KJ Vlassara H Cerami A Cachectin/tumour necrosis factor. Lancet (1989) 1:1122–6. 10.1016/S0140-6736(89)92394-5 2566060

[7] Luettig B Decker T Lohmann-Matthes ML. Evidence for the existence of two forms of membrane tumor necrosis factor: an integral protein and a molecule attached to its receptor. J Immunol (1989) 143:4034–8. 2556474

[8] Moss ML Jin SL Milla ME Bickett DM Burkhart W Carter HL Cloning of a disintegrin metalloproteinase that processes precursor tumour-necrosis factor-alpha. Nature (1997) 385:733–6. 10.1038/385733a0 9034191

[9] Eissner G Kolch W Scheurich P. Ligands working as receptors: reverse signaling by members of the TNF superfamily enhance the plasticity of the immune system. Cytokine Growth Factor Rev (2004) 15:353–66. 10.1016/j.cytogfr.2004.03.011 15450251

[10] Zandi E Rothwarf DM Delhase M Hayakawa M Karin M. The IkappaB kinase complex (IKK) contains two kinase subunits, IKKalpha and IKKbeta, necessary for IkappaB phosphorylation and NF-kappaB activation. Cell (1997) 91:243–52. 10.1016/S0092-8674(00)80406-7 9346241

[11] Bonizzi G Karin M The two NF-kappaB activation pathways and their role in innate and adaptive immunity. Trends Immunol (2004) 25:280–8. 10.1016/j.it.2004.03.008 15145317

[12] Kaltschmidt B Baeuerle PA Kaltschmidt C. Potential involvement of the transcription factor NF-kappa B in neurological disorders. Mol Aspects Med (1993) 14:171–90. 10.1016/0098-2997(93)90004-W 8264332

[13] Beutler B Krochin N Milsark IW Luedke C Cerami A. Control of cachectin (tumor necrosis factor) synthesis: mechanisms of endotoxin resistance. Science (1986) 232:977–80. 10.1126/science.3754653 3754653

[14] Naismith JH Devine TQ Brandhuber BJ Sprang SR. Crystallographic evidence for dimerization of unliganded tumor necrosis factor receptor. J Biol Chem (1995) 270:13303–7. 10.1074/jbc.270.22.13303 7768931

[15] Ihnatko R Kubes M. TNF signaling: early events and phosphorylation. Gen Physiol Biophys (2007) 26:159–67. 18063842

[16] Dopp JM Sarafian TA Spinella FM Kahn MA Shau H de Vellis J. Expression of the p75 TNF receptor is linked to TNF-induced NFkappaB translocation and oxyradical neutralization in glial cells. Neurochem Res (2002) 27:1535–42. 10.1023/A:1021608724117 12512958

[17] Grell M Douni E Wajant H Lohden M Clauss M Maxeiner B The transmembrane form of tumor necrosis factor is the prime activating ligand of the 80 kDa tumor necrosis factor receptor. Cell (1995) 83:793–802. 10.1016/0092-8674(95)90192-2 8521496

[18] Krippner-Heidenreich A Tubing F Bryde S Willi S Zimmermann G Scheurich P. Control of receptor-induced signaling complex formation by the kinetics of ligand/receptor interaction. J Biol Chem (2002) 277:44155–63. 10.1074/jbc.M207399200 12215450

[19] Waetzig GH Rosenstiel P Arlt A Till A Brautigam K Schafer H Soluble tumor necrosis factor (TNF) receptor-1 induces apoptosis via reverse TNF signaling and autocrine transforming growth factor-beta1. FASEB J (2005) 19:91–3. 10.1096/fj.04-2073fje 15514103

[20] Sabio G Davis RJ. TNF and MAP kinase signalling pathways. Semin Immunol (2014) 26:237–45. 10.1016/j.smim.2014.02.009 24647229 PMC4099309

[21] Guma M Firestein GS. c-Jun N-terminal kinase in inflammation and rheumatic diseases. Open Rheumatol J (2012) 6:220–31. 10.2174/1874312901206010220 23028407 PMC3460413

[22] Yang Y Kim SC Yu T Yi YS Rhee MH Sung GH Functional roles of p38 mitogen-activated protein kinase in macrophage-mediated inflammatory responses. Mediators Inflamm (2014) 2014:352371. 10.1155/2014/352371 24771982 PMC3977509

[23] Wilson NS Dixit V Ashkenazi A. Death receptor signal transducers: nodes of coordination in immune signaling networks. Nat Immunol (2009) 10:348–55. 10.1038/ni.1714 19295631

[24] Vanden BT Linkermann A Jouan-Lanhouet S Walczak H Vandenabeele P. Regulated necrosis: the expanding network of non-apoptotic cell death pathways. Nat Rev Mol Cell Biol (2014) 15:135–47. 10.1038/nrm3737 24452471

[25] Naude PJ den Boer JA Luiten PG Eisel UL. Tumor necrosis factor receptor cross-talk. FEBS J (2011) 278:888–98. 10.1111/j.1742-4658.2011.08017.x 21232019

[26] Sun SC Ley SC. New insights into NF-kappaB regulation and function. Trends Immunol (2008) 29:469–78. 10.1016/j.it.2008.07.003 18775672 PMC5751948

[27] Rauert H Wicovsky A Muller N Siegmund D Spindler V Waschke J Membrane tumor necrosis factor (TNF) induces p100 processing via TNF receptor-2 (TNFR2). J Biol Chem (2010) 285:7394–404. 10.1074/jbc.M109.037341 20038584 PMC2844188

[28] Fischer R Wajant H Kontermann R Pfizenmaier K Maier O. Astrocyte-specific activation of TNFR2 promotes oligodendrocyte maturation by secretion of leukemia inhibitory factor. Glia (2014) 62:272–83. 10.1002/glia.22605 24310780

[29] Fischer R Maier O Siegemund M Wajant H Scheurich P Pfizenmaier K. A TNF receptor 2 selective agonist rescues human neurons from oxidative stress-induced cell death. PLoS One (2011) 6:e27621. 10.1371/journal.pone.0027621 22110694 PMC3215731

[30] Zhang R Xu Y Ekman N Wu Z Wu J Alitalo K Etk/Bmx transactivates vascular endothelial growth factor 2 and recruits phosphatidylinositol 3-kinase to mediate the tumor necrosis factor-induced angiogenic pathway. J Biol Chem (2003) 278:51267–76. 10.1074/jbc.M310678200 14532277

[31] Zhou Z Gengaro P Wang W Wang XQ Li C Faubel S Role of NF-kappaB and PI 3-kinase/Akt in TNF-alpha-induced cytotoxicity in microvascular endothelial cells. Am J Physiol Renal Physiol (2008) 295:F932–41. 10.1152/ajprenal.00066.2008 18632801

[32] Rothe J Lesslauer W Lotscher H Lang Y Koebel P Kontgen F Mice lacking the tumour necrosis factor receptor 1 are resistant to TNF-mediated toxicity but highly susceptible to infection by *Listeria monocytogenes*. Nature (1993) 364:798–802. 10.1038/364798a0 8395024

[33] Musicki K Briscoe H Tran S Britton WJ Saunders BM. Differential requirements for soluble and transmembrane tumor necrosis factor in the immunological control of primary and secondary *Listeria monocytogenes* infection. Infect Immun (2006) 74:3180–9. 10.1128/IAI.02004-05 16714545 PMC1479262

[34] Torres D Janot L Quesniaux VF Grivennikov SI Maillet I Sedgwick JD Membrane tumor necrosis factor confers partial protection to *Listeria* infection. Am J Pathol (2005) 167:1677–87. 10.1016/S0002-9440(10)61250-3 16314479 PMC1613203

[35] Kontoyiannis D Pasparakis M Pizarro TT Cominelli F Kollias G. Impaired on/off regulation of TNF biosynthesis in mice lacking TNF AU-rich elements: implications for joint and gut-associated immunopathologies. Immunity (1999) 10:387–98. 10.1016/S1074-7613(00)80038-2 10204494

[36] Piguet PF Grau GE Vesin C Loetscher H Gentz R Lesslauer W. Evolution of collagen arthritis in mice is arrested by treatment with anti-tumour necrosis factor (TNF) antibody or a recombinant soluble TNF receptor. Immunology (1992) 77:510–4. 1337334 PMC1421669

[37] Mori L Iselin S De Libero G Lesslauer W. Attenuation of collagen-induced arthritis in 55-kDa TNF receptor type 1 (TNFR1)-IgG1-treated and TNFR1-deficient mice. J Immunol (1996) 157:3178–82. 8816431

[38] Probert L Akassoglou K Pasparakis M Kontogeorgos G Kollias G. Spontaneous inflammatory demyelinating disease in transgenic mice showing central nervous system-specific expression of tumor necrosis factor alpha. Proc Natl Acad Sci U S A (1995) 92:11294–8. 10.1073/pnas.92.24.11294 7479982 PMC40618

[39] Kassiotis G Kollias G. Uncoupling the proinflammatory from the immunosuppressive properties of tumor necrosis factor (TNF) at the p55 TNF receptor level: implications for pathogenesis and therapy of autoimmune demyelination. J Exp Med (2001) 193:427–34. 10.1084/jem.193.4.427 11181695 PMC2195909

[40] Barton A John S Ollier WE Silman A Worthington J Association between rheumatoid arthritis and polymorphism of tumor necrosis factor receptor II, but not tumor necrosis factor receptor I, in Caucasians. Arthritis Rheum (2001) 44:61–5. 10.1002/1529-0131(200101)44:1<61::AID-ANR9>3.0.CO;2-Q 11212177

[41] Dieude P Petit E Cailleau-Moindrault S Osorio J Pierlot C Martinez M Association between tumor necrosis factor receptor II and familial, but not sporadic, rheumatoid arthritis: evidence for genetic heterogeneity. Arthritis Rheum (2002) 46:2039–44. 10.1002/art.10101 12209506

[42] Orozco G Abelson AK Gonzalez-Gay MA Balsa A Pascual-Salcedo D Garcia A Study of functional variants of the BANK1 gene in rheumatoid arthritis. Arthritis Rheum (2009) 60:372–9. 10.1002/art.24244 19180476

[43] Sashio H Tamura K Ito R Yamamoto Y Bamba H Kosaka T Polymorphisms of the TNF gene and the TNF receptor superfamily member 1B gene are associated with susceptibility to ulcerative colitis and Crohn’s disease, respectively. Immunogenetics (2002) 53:1020–7. 10.1007/s00251-001-0423-7 11904678

[44] Komata T Tsuchiya N Matsushita M Hagiwara K Tokunaga K. Association of tumor necrosis factor receptor 2 (TNFR2) polymorphism with susceptibility to systemic lupus erythematosus. Tissue Antigens (1999) 53:527–33. 10.1034/j.1399-0039.1999.530602.x 10395102

[45] Chatzikyriakidou A Georgiou I Voulgari PV Drosos AA. The role of tumor necrosis factor (TNF)-alpha and TNF receptor polymorphisms in susceptibility to ankylosing spondylitis. Clin Exp Rheumatol (2009) 27:645–8. 19772798

[46] Pierik M Vermeire S Steen KV Joossens S Claessens G Vlietinck R Tumour necrosis factor-alpha receptor 1 and 2 polymorphisms in inflammatory bowel disease and their association with response to infliximab. Aliment Pharmacol Ther (2004) 20:303–10. 10.1111/j.1365-2036.2004.01946.x 15274667

[47] Ishikawa Y Kashiwase K Akaza T Morishima Y Inoko H Sasazuki T Polymorphisms in TNFA and TNFR2 affect outcome of unrelated bone marrow transplantation. Bone Marrow Transplant (2002) 29:569–75. 10.1038/sj.bmt.1703409 11979305

[48] Till A Rosenstiel P Krippner-Heidenreich A Mascheretti-Croucher S Croucher PJ Schafer H The Met-196 -> Arg variation of human tumor necrosis factor receptor 2 (TNFR2) affects TNF-alpha-induced apoptosis by impaired NF-kappaB signaling and target gene expression. J Biol Chem (2005) 280:5994–6004. 10.1074/jbc.M411541200 15572357

[49] Fontaine V Mohand-Said S Hanoteau N Fuchs C Pfizenmaier K Eisel U. Neurodegenerative and neuroprotective effects of tumor necrosis factor (TNF) in retinal ischemia: opposite roles of TNF receptor 1 and TNF receptor 2. J Neurosci (2002) 22:C216. 11917000 10.1523/JNEUROSCI.22-07-j0001.2002 PMC6758303

[50] Arnett HA Mason J Marino M Suzuki K Matsushima GK Ting JP. TNF alpha promotes proliferation of oligodendrocyte progenitors and remyelination. Nat Neurosci (2001) 4:1116–22. 10.1038/nn738 11600888

[51] McCoy MK Tansey MG. TNF signaling inhibition in the CNS: implications for normal brain function and neurodegenerative disease. J Neuroinflammation (2008) 5:45. 10.1186/1742-2094-5-45 18925972 PMC2577641

[52] Ban L Zhang J Wang L Kuhtreiber W Burger D Faustman DL. Selective death of autoreactive T cells in human diabetes by TNF or TNF receptor 2 agonism. Proc Natl Acad Sci U S A (2008) 105:13644–9. 10.1073/pnas.0803429105 18755894 PMC2533243

[53] Wang M Crisostomo PR Markel TA Wang Y Meldrum DR. Mechanisms of sex differences in TNFR2-mediated cardioprotection. Circulation (2008) 118:S38–45. 10.1161/CIRCULATIONAHA.107.756890 18824767 PMC2655136

[54] Rodriguez M Zoecklein L Papke L Gamez J Denic A Macura S Tumor necrosis factor alpha is reparative via TNFR2 [corrected] in the hippocampus and via TNFR1 [corrected] in the striatum after virus-induced encephalitis. Brain Pathol (2009) 19:12–26. 10.1111/j.1750-3639.2008.00151.x 18422761 PMC2613691

[55] Sakaguchi S. Naturally arising Foxp3-expressing CD25+CD4+ regulatory T cells in immunological tolerance to self and non-self. Nat Immunol (2005) 6:345–52. 10.1038/ni1178 15785760

[56] Zheng SG Wang J Wang P Gray JD Horwitz DA. IL-2 is essential for TGF-beta to convert naive CD4+CD25- cells to CD25+Foxp3+ regulatory T cells and for expansion of these cells. J Immunol (2007) 178:2018–27. 10.4049/jimmunol.178.4.2018 17277105

[57] Davidson TS DiPaolo RJ Andersson J Shevach EM. Cutting edge: IL-2 is essential for TGF-beta-mediated induction of Foxp3+ T regulatory cells. J Immunol (2007) 178:4022–6. 10.4049/jimmunol.178.7.4022 17371955

[58] Xu L Kitani A Fuss I Strober W. Cutting edge: regulatory T cells induce CD4+CD25-Foxp3- T cells or are self-induced to become Th17 cells in the absence of exogenous TGF-beta. J Immunol (2007) 178:6725–9. 10.4049/jimmunol.178.11.6725 17513718

[59] Wang P Zheng SG. Regulatory T cells and B cells: implication on autoimmune diseases. Int J Clin Exp Pathol (2013) 6:2668–74. 24294353 PMC3843247

[60] Nie J Li YY Zheng SG Tsun A Li B. FOXP3(+) Treg cells and gender bias in autoimmune diseases. Front Immunol (2015) 6:493. 10.3389/fimmu.2015.00493 26441996 PMC4585344

[61] Herman AE Freeman GJ Mathis D Benoist C. CD4+CD25+ T regulatory cells dependent on ICOS promote regulation of effector cells in the prediabetic lesion. J Exp Med (2004) 199:1479–89. 10.1084/jem.20040179 15184501 PMC2211778

[62] Lan Q Zhou X Fan H Chen M Wang J Ryffel B Polyclonal CD4+Foxp3+ Treg cells induce TGFbeta-dependent tolerogenic dendritic cells that suppress the murine lupus-like syndrome. J Mol Cell Biol (2012) 4:409–19. 10.1093/jmcb/mjs040 22773728 PMC3523557

[63] Eller K Wolf D Huber JM Metz M Mayer G McKenzie AN IL-9 production by regulatory T cells recruits mast cells that are essential for regulatory T cell-induced immune suppression. J Immunol (2011) 186:83–91. 10.4049/jimmunol.1001183 21115728 PMC3227733

[64] Fontenot JD Gavin MA Rudensky AY Pillars article: Foxp3 programs the development and function of CD4+CD25+ regulatory T cells. Nat. Immunol. 2003. 4: 330–336. J Immunol (2017) 198:986–92. 28115587

[65] Kim JM Rasmussen JP Rudensky AY. Regulatory T cells prevent catastrophic autoimmunity throughout the lifespan of mice. Nat Immunol (2007) 8:191–7. 10.1038/ni1428 17136045

[66] Annunziato F Cosmi L Liotta F Lazzeri E Manetti R Vanini V Phenotype, localization, and mechanism of suppression of CD4(+)CD25(+) human thymocytes. J Exp Med (2002) 196:379–87. 10.1084/jem.20020110 12163566 PMC2193942

[67] Chen X Baumel M Mannel DN Howard OM Oppenheim JJ. Interaction of TNF with TNF receptor type 2 promotes expansion and function of mouse CD4+CD25+ T regulatory cells. J Immunol (2007) 179:154–61. 10.4049/jimmunol.179.1.154 17579033

[68] Grinberg-Bleyer Y Saadoun D Baeyens A Billiard F Goldstein JD Gregoire S Pathogenic T cells have a paradoxical protective effect in murine autoimmune diabetes by boosting Tregs. J Clin Invest (2010) 120:4558–68. 10.1172/JCI42945 21099113 PMC2993590

[69] Chen X Wu X Zhou Q Howard OM Netea MG Oppenheim JJ. TNFR2 is critical for the stabilization of the CD4+Foxp3+ regulatory T cell phenotype in the inflammatory environment. J Immunol (2013) 190:1076–84. 10.4049/jimmunol.1202659 23277487 PMC3552130

[70] Chen X Oppenheim JJ. TNF-alpha: an activator of CD4+FoxP3+TNFR2+ regulatory T cells. Curr Dir Autoimmun (2010) 11:119–34. 10.1159/000289201 20173391 PMC6314650

[71] Nagar M Jacob-Hirsch J Vernitsky H Berkun Y Ben-Horin S Amariglio N TNF activates a NF-kappaB-regulated cellular program in human CD45RA-regulatory T cells that modulates their suppressive function. J Immunol (2010) 184:3570–81. 10.4049/jimmunol.0902070 20181891

[72] Chee J Angstetra E Mariana L Graham KL Carrington EM Bluethmann H TNF receptor 1 deficiency increases regulatory T cell function in nonobese diabetic mice. J Immunol (2011) 187:1702–12. 10.4049/jimmunol.1100511 21734073

[73] Kohm AP Carpentier PA Anger HA Miller SD. Cutting edge: CD4+CD25+ regulatory T cells suppress antigen-specific autoreactive immune responses and central nervous system inflammation during active experimental autoimmune encephalomyelitis. J Immunol (2002) 169:4712–6. 10.4049/jimmunol.169.9.4712 12391178

[74] Setoguchi R Hori S Takahashi T Sakaguchi S. Homeostatic maintenance of natural Foxp3(+) CD25(+) CD4(+) regulatory T cells by interleukin (IL)-2 and induction of autoimmune disease by IL-2 neutralization. J Exp Med (2005) 201:723–35. 10.1084/jem.20041982 15753206 PMC2212841

[75] Zaragoza B Chen X Oppenheim JJ Baeyens A Gregoire S Chader D Suppressive activity of human regulatory T cells is maintained in the presence of TNF. Nat Med (2016) 22:16–7. 10.1038/nm.4019 26735402 PMC6345394

[76] Nguyen DX Ehrenstein MR. Anti-TNF drives regulatory T cell expansion by paradoxically promoting membrane TNF-TNF-RII binding in rheumatoid arthritis. J Exp Med (2016) 213:1241–53. 10.1084/jem.20151255 27270893 PMC4925013

[77] Mahmud SA Manlove LS Schmitz HM Xing Y Wang Y Owen DL Costimulation via the tumor-necrosis factor receptor superfamily couples TCR signal strength to the thymic differentiation of regulatory T cells. Nat Immunol (2014) 15:473–81. 10.1038/ni.2849 24633226 PMC4000541

[78] Miller PG Bonn MB McKarns SC. Transmembrane TNF-TNFR2 impairs Th17 differentiation by promoting Il2 expression. J Immunol (2015) 195:2633–47. 10.4049/jimmunol.1500286 26268655 PMC4841279

[79] Chen X Subleski JJ Kopf H Howard OM Mannel DN Oppenheim JJ. Cutting edge: expression of TNFR2 defines a maximally suppressive subset of mouse CD4+CD25+FoxP3+ T regulatory cells: applicability to tumor-infiltrating T regulatory cells. J Immunol (2008) 180:6467–71. 10.4049/jimmunol.180.10.6467 18453563 PMC2699949

[80] Zheng SG Gray JD Ohtsuka K Yamagiwa S Horwitz DA. Generation ex vivo of TGF-beta-producing regulatory T cells from CD4+CD25- precursors. J Immunol (2002) 169:4183–9. 10.4049/jimmunol.169.8.4183 12370347

[81] Zheng SG Wang JH Gray JD Soucier H Horwitz DA. Natural and induced CD4+CD25+ cells educate CD4+CD25- cells to develop suppressive activity: the role of IL-2, TGF-beta, and IL-10. J Immunol (2004) 172:5213–21. 10.4049/jimmunol.172.9.5213 15100259

[82] Abbas AK Benoist C Bluestone JA Campbell DJ Ghosh S Hori S Regulatory T cells: recommendations to simplify the nomenclature. Nat Immunol (2013) 14:307–8. 10.1038/ni.2554 23507634

[83] Kanamori M Nakatsukasa H Okada M Lu Q Yoshimura A. Induced regulatory T cells: their development, stability, and applications. Trends Immunol (2016) 37:803–11. 10.1016/j.it.2016.08.012 27623114

[84] Li X Liang Y LeBlanc M Benner C Zheng Y. Function of a Foxp3 cis-element in protecting regulatory T cell identity. Cell (2014) 158:734–48. 10.1016/j.cell.2014.07.030 25126782 PMC4151505

[85] Okubo Y Mera T Wang L Faustman DL. Homogeneous expansion of human T-regulatory cells via tumor necrosis factor receptor 2. Sci Rep (2013) 3:3153. 10.1038/srep03153 24193319 PMC3818650

[86] Kong N Lan Q Su W Chen M Wang J Yang Z Induced T regulatory cells suppress osteoclastogenesis and bone erosion in collagen-induced arthritis better than natural T regulatory cells. Ann Rheum Dis (2012) 71:1567–72. 10.1136/annrheumdis-2011-201052 22764040 PMC4038329

[87] Su W Fan H Chen M Wang J Brand D He X Induced CD4+ forkhead box protein-positive T cells inhibit mast cell function and established contact hypersensitivity through TGF-beta1. J Allergy Clin Immunol (2012) 130:444–52. 10.1016/j.jaci.2012.05.011 22738679

[88] Zheng SG Wang J Horwitz DA. Cutting edge: Foxp3+CD4+CD25+ regulatory T cells induced by IL-2 and TGF-beta are resistant to Th17 conversion by IL-6. J Immunol (2008) 180:7112–6. 10.4049/jimmunol.180.11.7112 18490709

[89] Zhou X Kong N Wang J Fan H Zou H Horwitz D Cutting edge: all-trans retinoic acid sustains the stability and function of natural regulatory T cells in an inflammatory milieu. J Immunol (2010) 185:2675–9. 10.4049/jimmunol.1000598 20679534 PMC3098624

[90] Gu J Lu L Chen M Xu L Lan Q Li Q TGF-beta-induced CD4+Foxp3+ T cells attenuate acute graft-versus-host disease by suppressing expansion and killing of effector CD8+ cells. J Immunol (2014) 193:3388–97. 10.4049/jimmunol.1400207 25156367 PMC4247987

[91] Lan Q Fan H Quesniaux V Ryffel B Liu Z Zheng SG. Induced Foxp3(+) regulatory T cells: a potential new weapon to treat autoimmune and inflammatory diseases? J Mol Cell Biol (2012) 4:22–8. 10.1093/jmcb/mjr039 22107826 PMC3491614

[92] van Mierlo GJ Scherer HU Hameetman M Morgan ME Flierman R Huizinga TW Cutting edge: TNFR-shedding by CD4+CD25+ regulatory T cells inhibits the induction of inflammatory mediators. J Immunol (2008) 180:2747–51. 10.4049/jimmunol.180.5.2747 18292492

[93] Xanthoulea S Pasparakis M Kousteni S Brakebusch C Wallach D Bauer J Tumor necrosis factor (TNF) receptor shedding controls thresholds of innate immune activation that balance opposing TNF functions in infectious and inflammatory diseases. J Exp Med (2004) 200:367–76. 10.1084/jem.20040435 15289505 PMC2211976

[94] Engelmann H Holtmann H Brakebusch C Avni YS Sarov I Nophar Y Antibodies to a soluble form of a tumor necrosis factor (TNF) receptor have TNF-like activity. J Biol Chem (1990) 265:14497–504. 1696947

[95] Valencia X Stephens G Goldbach-Mansky R Wilson M Shevach EM Lipsky PE. TNF downmodulates the function of human CD4+CD25hi T-regulatory cells. Blood (2006) 108:253–61. 10.1182/blood-2005-11-4567 16537805 PMC1895836

[96] Ehrenstein MR Evans JG Singh A Moore S Warnes G Isenberg DA Compromised function of regulatory T cells in rheumatoid arthritis and reversal by anti-TNFalpha therapy. J Exp Med (2004) 200:277–85. 10.1084/jem.20040165 15280421 PMC2211983

[97] Stoop JN Woltman AM Biesta PJ Kusters JG Kuipers EJ Janssen HL Tumor necrosis factor alpha inhibits the suppressive effect of regulatory T cells on the hepatitis B virus-specific immune response. Hepatology (2007) 46:699–705. 10.1002/hep.21761 17654744

[98] Nie H Zheng Y Li R Guo TB He D Fang L Phosphorylation of FOXP3 controls regulatory T cell function and is inhibited by TNF-alpha in rheumatoid arthritis. Nat Med (2013) 19:322–8. 10.1038/nm.3085 23396208

[99] Nie H Zheng Y Li R Zhang J Reply to suppressive activity of human regulatory T cells is maintained in the presence of TNF. Nat Med (2016) 22:18–9. 10.1038/nm.4018 26735403

[100] Boschetti G Nancey S Sardi F Roblin X Flourie B Kaiserlian D Therapy with anti-TNFalpha antibody enhances number and function of Foxp3(+) regulatory T cells in inflammatory bowel diseases. Inflamm Bowel Dis (2011) 17:160–70. 10.1002/ibd.21308 20848510

[101] Toubi E Kessel A Mahmudov Z Hallas K Rozenbaum M Rosner I. Increased spontaneous apoptosis of CD4+CD25+ T cells in patients with active rheumatoid arthritis is reduced by infliximab. Ann N Y Acad Sci (2005) 1051:506–14. 10.1196/annals.1361.095 16126991

[102] Wu AJ Hua H Munson SH McDevitt HO. Tumor necrosis factor-alpha regulation of CD4+CD25+ T cell levels in NOD mice. Proc Natl Acad Sci U S A (2002) 99:12287–92. 10.1073/pnas.172382999 12221281 PMC129437

[103] Kontermann RE Scheurich P Pfizenmaier K. Antagonists of TNF action: clinical experience and new developments. Expert Opin Drug Discov (2009) 4:279–92. 10.1517/17460440902785167 23489126

[104] Cessak G Kuzawinska O Burda A Lis K Wojnar M Mirowska-Guzel D TNF inhibitors – mechanisms of action, approved and off-label indications. Pharmacol Rep (2014) 66:836–44. 10.1016/j.pharep.2014.05.004 25149988

[105] Pfizenmaier K Szymkowski DE Workshop summary: introduction to rational design of new means for therapeutic modulation of function of the TNF family. Adv Exp Med Biol (2011) 691:487–91. 10.1007/978-1-4419-6612-4_50 21153353

[106] Sedger LM McDermott MF TNF and TNF-receptors: from mediators of cell death and inflammation to therapeutic giants – past, present and future. Cytokine Growth Factor Rev (2014) 25:453–72. 10.1016/j.cytogfr.2014.07.016 25169849

[107] Monaco C Nanchahal J Taylor P Feldmann M. Anti-TNF therapy: past, present and future. Int Immunol (2015) 27:55–62. 10.1093/intimm/dxu102 25411043 PMC4279876

[108] Slifman NR Gershon SK Lee JH Edwards ET Braun MM. *Listeria monocytogenes* infection as a complication of treatment with tumor necrosis factor alpha-neutralizing agents. Arthritis Rheum (2003) 48:319–24. 10.1002/art.10758 12571839

[109] Sicotte NL Voskuhl RR. Onset of multiple sclerosis associated with anti-TNF therapy. Neurology (2001) 57:1885–8. 10.1212/WNL.57.10.1885 11723281

[110] Brown SL Greene MH Gershon SK Edwards ET Braun MM. Tumor necrosis factor antagonist therapy and lymphoma development: twenty-six cases reported to the food and drug administration. Arthritis Rheum (2002) 46:3151–8. 10.1002/art.10679 12483718

[111] Shakoor N Michalska M Harris CA Block JA. Drug-induced systemic lupus erythematosus associated with etanercept therapy. Lancet (2002) 359:579–80. 10.1016/S0140-6736(02)07714-0 11867114

[112] van Oosten BW Barkhof F Truyen L Boringa JB Bertelsmann FW von Blomberg BM Increased MRI activity and immune activation in two multiple sclerosis patients treated with the monoclonal anti-tumor necrosis factor antibody cA2. Neurology (1996) 47:1531–4. 10.1212/WNL.47.6.1531 8960740

[113] TNF neutralization in MS: results of a randomized, placebo-controlled multicenter study. The Lenercept Multiple Sclerosis Study Group and The University of British Columbia MS/MRI analysis group. Neurology (1999) 53:457–65. 10.1212/WNL.53.3.457 10449104

[114] Ruuls SR Hoek RM Ngo VN McNeil T Lucian LA Janatpour MJ Membrane-bound TNF supports secondary lymphoid organ structure but is subservient to secreted TNF in driving autoimmune inflammation. Immunity (2001) 15:533–43. 10.1016/S1074-7613(01)00215-1 11672536

[115] Saunders BM Tran S Ruuls S Sedgwick JD Briscoe H Britton WJ. Transmembrane TNF is sufficient to initiate cell migration and granuloma formation and provide acute, but not long-term, control of *Mycobacterium tuberculosis* infection. J Immunol (2005) 174:4852–9. 10.4049/jimmunol.174.8.4852 15814712

[116] Alexopoulou L Kranidioti K Xanthoulea S Denis M Kotanidou A Douni E Transmembrane TNF protects mutant mice against intracellular bacterial infections, chronic inflammation and autoimmunity. Eur J Immunol (2006) 36:2768–80. 10.1002/eji.200635921 16983719

[117] Fremond C Allie N Dambuza I Grivennikov SI Yeremeev V Quesniaux VF Membrane TNF confers protection to acute mycobacterial infection. Respir Res (2005) 6:136. 10.1186/1465-9921-6-136 16285886 PMC1325056

[118] Steed PM Tansey MG Zalevsky J Zhukovsky EA Desjarlais JR Szymkowski DE Inactivation of TNF signaling by rationally designed dominant-negative TNF variants. Science (2003) 301:1895–8. 10.1126/science.1081297 14512626

[119] Zalevsky J Secher T Ezhevsky SA Janot L Steed PM O’Brien C Dominant-negative inhibitors of soluble TNF attenuate experimental arthritis without suppressing innate immunity to infection. J Immunol (2007) 179:1872–83. 10.4049/jimmunol.179.3.1872 17641054

[120] Olleros ML Vesin D Fotio AL Santiago-Raber ML Tauzin S Szymkowski DE Soluble TNF, but not membrane TNF, is critical in LPS-induced hepatitis. J Hepatol (2010) 53:1059–68. 10.1016/j.jhep.2010.05.029 20813418

[121] Taoufik E Tseveleki V Chu SY Tselios T Karin M Lassmann H Transmembrane tumour necrosis factor is neuroprotective and regulates experimental autoimmune encephalomyelitis via neuronal nuclear factor-kappaB. Brain (2011) 134:2722–35. 10.1093/brain/awr203 21908876

[122] Brambilla R Ashbaugh JJ Magliozzi R Dellarole A Karmally S Szymkowski DE Inhibition of soluble tumour necrosis factor is therapeutic in experimental autoimmune encephalomyelitis and promotes axon preservation and remyelination. Brain (2011) 134:2736–54. 10.1093/brain/awr199 21908877 PMC3170538

[123] Novrup HG Bracchi-Ricard V Ellman DG Ricard J Jain A Runko E Central but not systemic administration of XPro1595 is therapeutic following moderate spinal cord injury in mice. J Neuroinflammation (2014) 11:159 10.1186/s12974-014-0159-6 25204558 PMC4176557

[124] Shibata H Yoshioka Y Ohkawa A Minowa K Mukai Y Abe Y Creation and X-ray structure analysis of the tumor necrosis factor receptor-1-selective mutant of a tumor necrosis factor-alpha antagonist. J Biol Chem (2008) 283:998–1007. 10.1074/jbc.M707933200 18003610

[125] Shibata H Yoshioka Y Ohkawa A Abe Y Nomura T Mukai Y The therapeutic effect of TNFR1-selective antagonistic mutant TNF-alpha in murine hepatitis models. Cytokine (2008) 44:229–33. 10.1016/j.cyto.2008.07.003 18815054

[126] Nomura T Abe Y Kamada H Shibata H Kayamuro H Inoue M Therapeutic effect of PEGylated TNFR1-selective antagonistic mutant TNF in experimental autoimmune encephalomyelitis mice. J Control Release (2011) 149:8–14. 10.1016/j.jconrel.2009.12.015 20036293

[127] McCann FE Perocheau DP Ruspi G Blazek K Davies ML Feldmann M Selective tumor necrosis factor receptor I blockade is antiinflammatory and reveals immunoregulatory role of tumor necrosis factor receptor II in collagen-induced arthritis. Arthritis Rheumatol (2014) 66:2728–38. 10.1002/art.38755 24965881

[128] Taoufik E Petit E Divoux D Tseveleki V Mengozzi M Roberts ML TNF receptor I sensitizes neurons to erythropoietin- and VEGF-mediated neuroprotection after ischemic and excitotoxic injury. Proc Natl Acad Sci U S A (2008) 105:6185–90. 10.1073/pnas.0801447105 18413601 PMC2299225

[129] Loetscher H Stueber D Banner D Mackay F Lesslauer W. Human tumor necrosis factor alpha (TNF alpha) mutants with exclusive specificity for the 55-kDa or 75-kDa TNF receptors. J Biol Chem (1993) 268:26350–7. 8253759

[130] Kretschmer K Apostolou I Jaeckel E Khazaie K von Boehmer H. Making regulatory T cells with defined antigen specificity: role in autoimmunity and cancer. Immunol Rev (2006) 212:163–9. 10.1111/j.0105-2896.2006.00411.x 16903913

[131] Fournel S Wieckowski S Sun W Trouche N Dumortier H Bianco A C3-symmetric peptide scaffolds are functional mimetics of trimeric CD40L. Nat Chem Biol (2005) 1:377–82. 10.1038/nchembio746 16370373

[132] Monden Y Kubota T Inoue T Tsutsumi T Kawano S Ide T Tumor necrosis factor-alpha is toxic via receptor 1 and protective via receptor 2 in a murine model of myocardial infarction. Am J Physiol Heart Circ Physiol (2007) 293:H743–53. 10.1152/ajpheart.00166.2007 17416608

[133] Rezzoug F Huang Y Tanner MK Wysoczynski M Schanie CL Chilton PM TNF-alpha is critical to facilitate hemopoietic stem cell engraftment and function. J Immunol (2008) 180:49–57. 10.4049/jimmunol.180.1.49 18097003

[134] Yong J Lacan G Dang H Hsieh T Middleton B Wasserfall C BCG vaccine-induced neuroprotection in a mouse model of Parkinson’s disease. PLoS One (2011) 6:e16610. 10.1371/journal.pone.0016610 21304945 PMC3031604

[135] Khan SQ Tsai MS Schreiber TH Wolf D Deyev VV Podack ER. Cloning, expression, and functional characterization of TL1A-Ig. J Immunol (2013) 190:1540–50. 10.4049/jimmunol.1201908 23319737

[136] Faustman DL Wang L Okubo Y Burger D Ban L Man G Proof-of-concept, randomized, controlled clinical trial of Bacillus-Calmette-Guerin for treatment of long-term type 1 diabetes. PLoS One (2012) 7:e41756. 10.1371/journal.pone.0041756 22905105 PMC3414482

[137] Ablamunits V Herold KC. Generation and function of human regulatory CD8+ T cells induced by a humanized OKT3 monoclonal antibody hOKT3gamma1 (Ala-Ala). Hum Immunol (2008) 69:732–6. 10.1016/j.humimm.2008.08.290 18817833

[138] Hegazy DM O’Reilly DA Yang BM Hodgkinson AD Millward BA Demaine AG. NFkappaB polymorphisms and susceptibility to type 1 diabetes. Genes Immun (2001) 2:304–8. 10.1038/sj.gene.6363776 11607785

[139] Carpentier I Coornaert B Beyaert R. Function and regulation of tumor necrosis factor receptor type 2. Curr Med Chem (2004) 11:2205–12. 10.2174/0929867043364694 15279559

[140] Douni E Kollias G. A critical role of the p75 tumor necrosis factor receptor (p75TNF-R) in organ inflammation independent of TNF, lymphotoxin alpha, or the p55TNF-R. J Exp Med (1998) 188:1343–52. 10.1084/jem.188.7.1343 9763613 PMC2212501

[141] Plenge RM Seielstad M Padyukov L Lee AT Remmers EF Ding B TRAF1-C5 as a risk locus for rheumatoid arthritis – a genomewide study. N Engl J Med (2007) 357:1199–209. 10.1056/NEJMoa073491 17804836 PMC2636867

[142] Welborn MR Van Zee K Edwards PD Pruitt JH Kaibara A Vauthey JN A human tumor necrosis factor p75 receptor agonist stimulates in vitro T cell proliferation but does not produce inflammation or shock in the baboon. J Exp Med (1996) 184:165–71. 10.1084/jem.184.1.165 8691130 PMC2192685

[143] Haxhinasto S Mathis D Benoist C. The AKT-mTOR axis regulates de novo differentiation of CD4+Foxp3+ cells. J Exp Med (2008) 205:565–74. 10.1084/jem.20071477 18283119 PMC2275380

